# The Gene *scb-1* Underlies Variation in *Caenorhabditis elegans* Chemotherapeutic Responses

**DOI:** 10.1534/g3.120.401310

**Published:** 2020-05-08

**Authors:** Kathryn S. Evans, Erik C. Andersen

**Affiliations:** * Molecular Biosciences, ^†^Northwestern University, Evanston, IL 60208; ^†^ Interdisciplinary Biological Sciences Program, Northwestern University, Evanston, IL 60208, and; ^‡^ Robert H. Lurie Comprehensive Cancer Center, Northwestern University, Chicago, IL 60611

**Keywords:** *C. elegans*, QTL, pleiotropy, chemotherapeutics, mediation

## Abstract

Pleiotropy, the concept that a single gene controls multiple distinct traits, is prevalent in most organisms and has broad implications for medicine and agriculture. The identification of the molecular mechanisms underlying pleiotropy has the power to reveal previously unknown biological connections between seemingly unrelated traits. Additionally, the discovery of pleiotropic genes increases our understanding of both genetic and phenotypic complexity by characterizing novel gene functions. Quantitative trait locus (QTL) mapping has been used to identify several pleiotropic regions in many organisms. However, gene knockout studies are needed to eliminate the possibility of tightly linked, non-pleiotropic loci. Here, we use a panel of 296 recombinant inbred advanced intercross lines of *Caenorhabditis elegans* and a high-throughput fitness assay to identify a single large-effect QTL on the center of chromosome V associated with variation in responses to eight chemotherapeutics. We validate this QTL with near-isogenic lines and pair genome-wide gene expression data with drug response traits to perform mediation analysis, leading to the identification of a pleiotropic candidate gene, *scb-1*, for some of the eight chemotherapeutics. Using deletion strains created by genome editing, we show that *scb-1*, which was previously implicated in response to bleomycin, also underlies responses to other double-strand DNA break-inducing chemotherapeutics. This finding provides new evidence for the role of *scb-1* in the nematode drug response and highlights the power of mediation analysis to identify causal genes.

Pleiotropy refers to the well established notion that a single gene or genetic variant affects multiple distinct traits (Paaby and Rockman 2013), and the discovery of pleiotropic genes can provide meaningful insights into the molecular mechanisms of these traits (Tyler *et al.* 2016). It has become easier to identify pleiotropic genes with the advent of reverse-genetic screens and quantitative trait locus (QTL) mapping (Paaby and Rockman 2013). For example, pleiotropic QTL for diverse growth and fitness traits have been identified in organisms such as yeast (Cubillos *et al.* 2011; Jerison *et al.* 2017; Peltier *et al.* 2019), *Arabidopsis* (McKay *et al.* 2003; El‐Assal *et al.* 2004; Fusari *et al.* 2017), *Drosophila* (Brown *et al.* 2013; McGuigan *et al.* 2014), and mice (White *et al.* 2013; Leamy *et al.* 2014; Lin *et al.* 2014). These studies have led to important questions in the field of evolutionary genetics regarding the ‘cost of complexity’ (Fisher; Orr 2000), as a single mutation might be beneficial for one trait and harmful for another (Wagner and Zhang 2011). Furthermore, human association studies have identified pleiotropic variants associated with different diseases (Sivakumaran *et al.* 2011; Pavlides *et al.* 2016; Chesmore *et al.* 2018), highlighting both the ubiquity and importance of certain immune-related genes and oncogenes across unrelated diseases (Borrello *et al.* 2008; Gratten and Visscher 2016). Perhaps the strongest evidence of pleiotropy exists for molecular phenotypes. Large-scale expression QTL (eQTL) mapping studies have identified single regulatory variants that control expression and likely the functions of hundreds of genes at once, opening a window into the mechanisms for how traits are controlled (Keurentjes *et al.* 2007; Breitling *et al.* 2008; Rockman *et al.* 2010; Albert and Kruglyak 2015; Hasin-Brumshtein *et al.* 2016; Albert *et al.* 2018).

The nematode *Caenorhabditis elegans* provides a tractable metazoan model to identify and study pleiotropic QTL (Paaby and Rockman 2013). A large panel of recombinant inbred advanced intercross lines (RIAILs) derived from two divergent strains, N2 and CB4856 (Rockman and Kruglyak 2009; Andersen *et al.* 2015), has been leveraged in several linkage mapping analyses (Li *et al.* 2006; Gutteling *et al.* 2007b, 2007a; Kammenga *et al.* 2007; Seidel *et al.* 2008, 2011; Reddy *et al.* 2009; McGrath *et al.* 2009; Doroszuk *et al.* 2009; Viñuela *et al.* 2010; Rockman *et al.* 2010; Bendesky *et al.* 2011, 2012; Bendesky and Bargmann 2011; Rodriguez *et al.* 2012; Andersen *et al.* 2014; Glater *et al.* 2014; Snoek *et al.* 2014; Balla *et al.* 2015; Schmid *et al.* 2015; Singh *et al.* 2016; Zdraljevic *et al.* 2017, 2019; Lee *et al.* 2017; Zamanian *et al.* 2018a; Evans *et al.* 2018; Brady *et al.* 2019). Quantitative genetic analysis using these panels and a high-throughput phenotyping assay (Andersen *et al.* 2015) has facilitated the discovery of numerous QTL (Zamanian *et al.* 2018b), several quantitative trait genes (QTG) (Brady *et al.* 2019) and quantitative trait nucleotides (QTN) (Zdraljevic *et al.* 2017, 2019) underlying fitness-related traits in the nematode. Additionally, three pleiotropic genomic regions were recently found to influence responses to a diverse group of toxins (Evans *et al.* 2018). However, overlapping genomic regions might not represent true pleiotropy but could demonstrate the co-existence of tightly linked loci (Paaby and Rockman 2013).

Here, we use linkage mapping to identify a single overlapping QTL on chromosome V that influences the responses to eight chemotherapeutic compounds. We show that these drug-response QTL also overlap with an expression QTL hotspot that contains the gene *scb-1*, previously implicated in bleomycin response (Brady *et al.* 2019). Although the exact mechanism of *scb-1* is yet unknown, it is hypothesized to act in response to stress (Riedel *et al.* 2013) and has weak homology to a viral hydrolase (Kelley *et al.* 2015; Zhang *et al.* 2018). Together, these data suggest that the importance of *scb-1* expression might extend beyond bleomycin response. We validated the QTL using near-isogenic lines (NILs) and performed mediation analysis to predict that *scb-1* expression explains the observed QTL for four of the eight drugs. Finally, we directly tested the effect of *scb-1* loss of function on chemotherapeutic responses. We discovered that expression of *scb-1* underlies differential responses to several chemotherapeutics that cause double-strand DNA breaks, not just bleomycin. This discovery of pleiotropy helps to further define the role of *scb-1* by expanding its known functions and provides insights into the molecular mechanisms underlying the nematode drug response.

## Materials and Methods

### Strains

Animals were grown at 20° on modified nematode growth media (NGMA) containing 1% agar and 0.7% agarose to prevent burrowing and fed OP50 (Ghosh *et al.* 2012). The two parental strains, the canonical laboratory strain, N2, and the wild isolate from Hawaii, CB4856, were used to generate all recombinant lines. 208 recombinant inbred advanced intercross lines (RIAILs) generated previously by Rockman *et al.* (Rockman and Kruglyak 2009) (set 1 RIAILs) were phenotyped for expression QTL mapping (detailed below). A second set of 296 RIAILs generated previously by Andersen *et al.* (Andersen *et al.* 2015) (set 2 RIAILs) was used more extensively for drug phenotyping and linkage mapping. The set 2 RIAILs were used for linkage mapping because they addressed the three main disadvantages of the set 1 RIAILs detailed previously (Andersen *et al.* 2015), namely a structured population, the laboratory-derived variant in *npr-1* (Sterken *et al.* 2015), and the *peel-1* *zeel-1* incompatibility (Seidel *et al.* 2008, 2011). Because of these limitations, the set 2 RIAILs were generated using QX1430 and CB4856. QX1430 is from the N2 strain background but contains a transposon insertion in *peel-1* and the CB4856 *npr-1* allele introgressed on chromosome X (Andersen *et al.* 2015). Near-isogenic lines (NILs) were generated by backcrossing a selected RIAIL for several generations to the parent strain (N2 or CB4856) (Brady *et al.* 2019) using PCR amplicons for insertion-deletion (indels) variants to track the introgressed region. NILs were whole-genome sequenced to verify introgressions were only in the targeted genomic intervals. CRISPR-Cas9-mediated deletions of *scb-1* were described previously (Brady *et al.* 2019). All strains are available upon request or from the *C. elegans* Natural Diversity Resource (Cook *et al.* 2017). Primers used to generate ECA1114 can be found in the Supplemental Information.

### High-throughput fitness assays for linkage mapping

For dose responses and RIAIL phenotyping, we used a high-throughput fitness assay described previously (Andersen *et al.* 2015). In summary, populations of each strain were passaged and amplified on NGMA plates for four generations. In the fifth generation, gravid adults were bleach-synchronized and 25-50 embryos from each strain were aliquoted into 96-well microtiter plates at a final volume of 50 µL K medium (Boyd *et al.* 2012). The following day, arrested L1s were fed HB101 bacterial lysate (Pennsylvania State University Shared Fermentation Facility, State College, PA; (García-González *et al.* 2017)) at a final concentration of 5 mg/mL in K medium and were grown to the L4 larval stage for 48 hr at 20° with constant shaking. Three L4 larvae were sorted into new 96-well microtiter plates containing 10 mg/mL HB101 bacterial lysate, 50 µM kanamycin, and either diluent (1% water or 1% DMSO) or drug dissolved in the diluent using a large-particle flow cytometer (COPAS BIOSORT, Union Biometrica; Holliston, MA). Sorted animals were grown for 96 hr at 20° with constant shaking. The next generation of animals and the parents were treated with sodium azide (50 mM in 1X M9) to straighten their bodies for more accurate length measurements. Animal length (median.TOF), optical density integrated over animal length (median.EXT), and brood size (norm.n) were quantified for each well using the COPAS BIOSORT. Nematodes get longer (animal length) and become thicker and more complex (optical density) over developmental time. Because animal length and optical density are highly correlated, we calculated a fourth trait (median.norm.EXT) that normalizes optical density by animal length (median.EXT / median.TOF). Phenotypic measurements collected by the BIOSORT were processed and analyzed using the R package *easysorter* (Shimko and Andersen 2014) as described previously (Brady *et al.* 2019). Differences among strains within the control conditions were controlled by subtracting the mean control-condition value from each drug-condition replicate for each strain using a linear model (*drug_phenotype ∼ mean_control_phenotype)*. In this way, we are addressing only the differences among strains that were caused by the drug condition and the variance in the control condition does not affect the variance in the drug condition. For plotting purposes, these residual values (negative and positive residuals) were normalized from 0 to 1 where 0 refers to the smallest residual phenotypic value in that condition and 1 refers to the largest.

### Dose-response assays

Four genetically divergent strains (N2, CB4856, JU258, and DL238) were treated with increasing concentrations of each of the eight drugs using the high-throughput fitness assay described above. The dose of each drug that provided a reproducible drug-specific effect that maximizes between-strain variation while minimizing within-strain variation across the four traits was selected for the linkage mapping experiments. The chosen concentrations are as follows: 100 µM amsacrine hydrochloride (Fisher Scientific, #A277720MG) in DMSO, 50 µM bleomycin sulfate (Fisher, #50-148-546) in water, 2 µM bortezomib (VWR, #AAJ60378-MA) in DMSO, 250 µM carmustine (Sigma, #1096724-75MG) in DMSO, 500 µM cisplatin (Sigma, #479306-1G) in K media, 500 µM etoposide (Sigma, #E1383) in DMSO, 500 µM puromycin dihydrochloride (VWR, #62111-170) in water, and 150 µM silver nitrate (Sigma-Aldrich, #S6506-5G) in water.

### Linkage mapping

Set 1 and set 2 RIAILs were phenotyped in each of the eight drugs and controls using the high-throughput fitness assay described above. Linkage mapping was performed on each of the drug and gene expression traits using the R package *linkagemapping* (https://github.com/AndersenLab/linkagemapping) as described previously (Brady *et al.* 2019). The cross object derived from the whole-genome sequencing of the RIAILs containing 13,003 SNPs was loaded using the function *load_cross_obj(“N2xCB4856cross_full”)*. The RIAIL phenotypes were merged into the cross object using the *merge_pheno* function with the argument *set = 1* for expression QTL mapping and *set = 2* for drug phenotype mapping. A forward search (*fsearch* function) adapted from the *R/qtl* package (Broman *et al.* 2003) was used to calculate the logarithm of the odds (LOD) scores for each genetic marker and each trait as *-n(ln(1-R^2^)/2ln(10))* where R is the Pearson correlation coefficient between the RIAIL genotypes at the marker and trait phenotypes (Bloom *et al.* 2013). A 5% genome-wide error rate was calculated by permuting the RIAIL phenotypes 1000 times. The marker with the highest LOD score above the significance threshold was selected as the QTL then integrated into the model as a cofactor and mapping was repeated iteratively until no further significant QTL were identified. Finally, the *annotate_lods* function was used to calculate the effect size of each QTL and determine 95% confidence intervals defined by a 1.5 LOD drop from the peak marker using the argument *cutoff = proximal*.

### Modified high-throughput fitness assay for NIL validation

NILs and *scb-1* deletion strains were tested using a modified version of the high-throughput fitness assay detailed above. Strains were propagated for two generations, bleach-synchronized in three independent replicates, and titered at a concentration of 25-50 embryos per well of a 96-well microtiter plate. The following day, arrested L1s were fed HB101 bacterial lysate at a final concentration of 5 mg/mL with either diluent or drug. After 48 hr of growth at 20° with constant shaking, nematodes were treated with sodium azide (5 mM in water) prior to analysis of animal length and optical density using the COPAS BIOSORT. As only one generation of growth is observed, brood size was not calculated. A single trait (median.EXT) was chosen to represent animal growth generally, as the trait is defined by integrating optical density over length. Because of the modified timing of the drug delivery, lower drug concentrations were needed to recapitulate the previously observed phenotypic effect. The selected doses are as follows: 12.5 µM amsacrine in DMSO, 12.5 µM bleomycin in water, 2 µM bortezomib in DMSO, 250 µM carmustine in DMSO, 125 µM cisplatin in K media, 62.5 µM etoposide in DMSO, 300 µM puromycin in water, and 100 µM silver in water.

### Expression QTL analysis

Microarray data for gene expression using 15,888 probes were previously collected from synchronized young adult populations of 208 set 1 RIAILs (Rockman *et al.* 2010). Expression data were corrected for dye effects and probes with variants were removed (Andersen *et al.* 2014). Linkage mapping was performed as described above for the remaining 14,107 probes, and a significance threshold was determined using a permutation-based False Discovery Rate (FDR). FDR was calculated as the ratio of the average number of genes across 10 permutations expected by chance to show a maximum LOD score greater than a particular threshold *vs.* the number of genes observed in the real data with a maximum LOD score greater than that threshold. We calculated the FDR for a range of thresholds from 2 to 10, with increasing steps of 0.01, and set the threshold so that the calculated FDR was less than 5%.

Local eQTL were defined as linkages whose peak LOD scores were within 1 Mb of the starting position of the probe (Rockman *et al.* 2010). eQTL hotspots were identified by dividing the genome into 5 cM bins and counting the number of distant eQTL that mapped to each bin. Significance was determined as bins with more eQTL than the Bonferroni-corrected 99^th^ percentile of a Poisson distribution with a mean of 3.91 QTL (total QTL / total bins) (Brem *et al.* 2002; Rockman *et al.* 2010; Evans *et al.* 2018). We identified nine eQTL hotspots (II, IVL, IVC, IVR, VL, VC, VR, XL, and XC). To avoid false positives, we increased the LOD threshold for QTL to be counted in the hotspot analysis to a LOD > 5 or LOD > 6. At a LOD > 5, six of the nine eQTL hotspots persist (IVL, IVR, VC, VR, XL, and XC), and at a LOD > 6, three persist (IVL, IVR, and XL). We further looked for spurious eQTL hotspots in ten permuted datasets. At a LOD > 5, we identified four hotspots, and at a LOD > 6, we identified one hotspot.

### Mediation analysis

A total of 159 set 1 RIAILs were phenotyped in each of the eight drugs and controls using the standard high-throughput fitness assay described above. Mediation scores were calculated with bootstrapping using the *mediate* function from the *mediation* R package (version 4.4.7) (Tingley *et al.* 2014) for each QTL identified from the set 1 RIAILs and all 49 probes (including *scb-1*, A_12_P104350) that mapped to the chromosome V eQTL hotspot using the following models:

(1) Mediator model:lm(expression∼genotype)

(2) Outcome model:lm(phenotype∼expression+genotype)

The output of the *mediate* function can be summarized as follows: the total effect of genotype on phenotype, ignoring expression (*tau.coef*); the direct effect of genotype on phenotype, while holding expression constant (*z0*); the estimated effect of expression on phenotype (*d0*); the proportion of the total effect that can be explained by expression data (*n0*). This mediation proportion (*n0*) can be a useful way to identify the impact of gene expression on the overall phenotype. However, cases of inconsistent mediation (where the direct effect is either smaller than or in the opposite direction of the indirect mediation effect) render this measurement uninterpretable with values greater than one or less than zero (MacKinnon *et al.* 2007). We used the estimated effect of expression on phenotype (*z0*) as the final mediation score for this reason. Because the effect size can be positive or negative, mediation scores range from -1 to 1, and we evaluated the absolute value of mediation estimates to compare across traits. Each mediation estimate generated a p-value, indicating confidence in the estimate, derived from bootstrapping with 1000 simulations. The likelihood of *scb-1* mediating a given QTL effect was calculated relative to the other 48 probes with an eQTL in the region (Table S1). Traits in which *scb-1* was at or above the 90^th^ percentile of this distribution were prioritized over other traits.

### Statistical analysis

Broad-sense heritability was calculated from the dose response phenotypes using the *lmer* function in the *lme4* R package (Bates *et al.* 2014) with the formula *phenotype ∼1 + (1|strain)* for each dose. For the NIL and *scb-1* deletion high-throughput assays, statistical significance of phenotypic differences between each strain pair was tested using the *TukeyHSD* function (R Core Team 2017) on an ANOVA model with the formula *phenotype ∼ strain* to assess differences between strains in the control-regressed phenotype data.

### Data availability

File S1 contains the results of the original dose response high-throughput fitness assay. File S2 contains the residual phenotypic values for all 159 set 1 RIAILs, 296 set 2 RIAILs, and parent strains (N2 and CB4856) in response to all eight chemotherapeutics. File S3 contains the linkage mapping results for the set 2 RIAILs for all 32 drug-response traits tested in the high-throughput fitness assay. File S4 is a VCF that reports the genotype of ECA1114. File S5 contains the simplified genotype of all the NILs in the study. File S6 contains the raw pruned phenotypes for the NIL dose response with the modified high-throughput fitness assay. File S7 contains the pairwise statistical significance for all strains and high-throughput assays. File S8 contains the microarray expression data for 14,107 probes from Rockman *et al.* 2010. File S9 contains the linkage mapping results for the expression data obtained with the set 1 RIAILs. File S10 contains the location of each eQTL hotspot and a list of genes with an eQTL in each hotspot. File S11 contains the linkage mapping results from the set 1 RIAILs for all 32 drug-response traits tested in the high-throughput fitness assay. File S12 contains the pairwise mediation estimates for all 32 drug-response traits and all 49 probes. File S13 contains the raw pruned phenotypes for the *scb-1* deletion modified high-throughput fitness assay. The datasets and code for generating figures can be found at https://github.com/AndersenLab/scb1_mediation_manuscript. Supplemental material available at figshare: https://doi.org/10.25387/g3.12250091.

## Results

### Natural variation on chromosome V underlies differences in responses to several chemotherapeutics

We measured *C. elegans* development and chemotherapeutic sensitivity as a function of animal length (median.TOF), optical density (median.EXT), and brood size (norm.n) with a high-throughput assay developed using the COPAS BIOSORT (see Methods) (Andersen *et al.* 2015; Zdraljevic *et al.* 2017, 2019; Evans *et al.* 2018; Brady *et al.* 2019). Animal length and optical density (animal thickness and composition) are both measures of nematode development, and brood size is a measure of nematode reproduction (Andersen *et al.* 2015). Because optical density is calculated as a function of length and these traits are related, a fourth trait that captures the optical density normalized by length (median.norm.EXT) was also included. We exposed four genetically divergent strains (N2, CB4856, JU258, and DL238) to increasing doses of eight chemotherapeutic compounds. Five of these compounds (bleomycin, carmustine, etoposide, amsacrine, and cisplatin) are known to cause double-strand DNA breaks and/or inhibit DNA synthesis (Dorr 1992; Ketron *et al.* 2012; Dasari and Tchounwou 2014; Montecucco *et al.* 2015; Nikolova *et al.* 2017). The remaining three compounds either inhibit protein synthesis (puromycin) (Azzam and Algranati 1973), inhibit the proteosome and subsequent protein degradation (bortezomib) (Piperdi *et al.* 2011), or cause cellular toxicity in a poorly defined way (silver nitrate) (Kaplan *et al.* 2016) (Table 1). In the presence of each drug, nematodes were generally shorter, less optically dense, and produced smaller broods compared to non-treated nematodes (Figure S1, File S1). We observed significant phenotypic variation among strains and identified a substantial heritable genetic component for most traits (average *H*^2^ = 0.52 +/− 0.53).

**Table 1:** Main mechanism of action for eight chemotherapeutic drugs

Drug	Drug class	Mechanism of action
Amsacrine	Topoisomerase inhibitors	DNA intercalation and inhibition of topoisomerase II, causing DNA double-strand breaks, cell cycle arrest, and cell death
Bleomycin	Antitumor antibiotic	Forms complexes with iron that reduce molecular oxygen to form free radicals which in turn cause DNA single- and double-strand breaks
Bortezomib	Proteosome inhibitors	Reversibly inhibits the 26S proteosome and inhibits nuclear factor (NF)-kappaB causing disruption of various cell signaling pathways, cell cycle arrest, and cell death.
Carmustine	Alkylating agents	Alkylates and cross-links DNA causing cell cycle arrest and cell death
Cisplatin	Alkylating agents	Alkylates and cross-links DNA causing cell cycle arrest and cell death
Etoposide	Topoisomerase inhibitors	Binds to and inhibits topoisomerase II causing an increase of DNA single- and double-strand breaks, cell cycle arrest, and cell death
Puromycin	Aminonucleoside antibiotic	Acts as analog of 3′ terminal end of aminoacyl-tRNA and incorporates itself into growing polypeptide chain causing premature termination and inhibition of protein synthesis
Silver	NA	Multi-faceted induction of apoptosis

**Figure 1:**
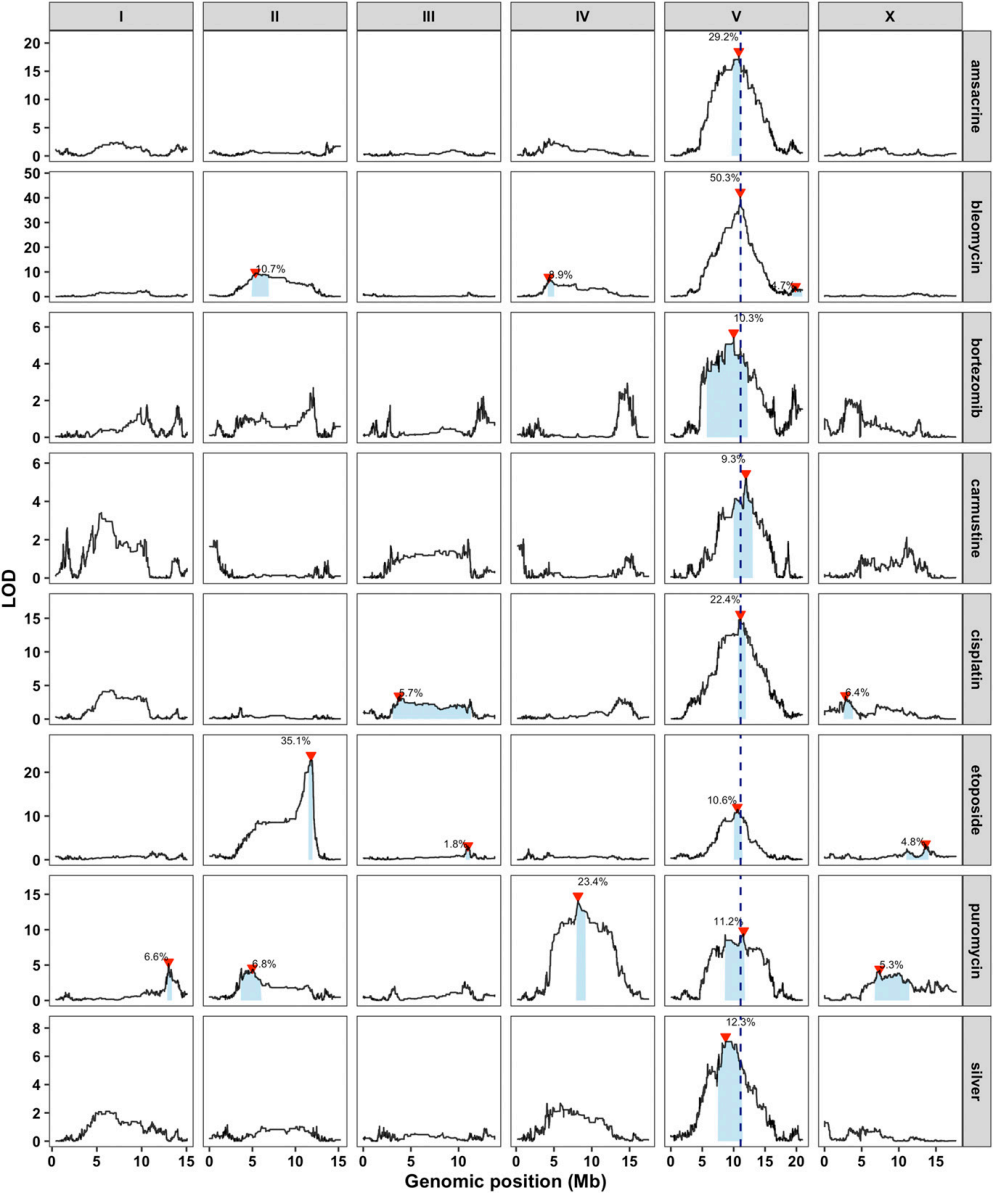
A large-effect QTL on the center of chromosome V underlies responses to several chemotherapeutics. Linkage mapping results with the set 2 RIAILs for a representative trait for each drug are shown (amascrine: median.norm.EXT, bleomycin median.TOF:, bortezomib: median.TOF, carmustine: norm.n, cisplatin: median.TOF, etoposide: norm.n, puromycin: median.TOF, silver: median.TOF). Genomic position (x-axis) is plotted against the logarithm of the odds (LOD) score (y-axis) for 13,003 genomic markers. Each significant QTL is indicated by a red triangle at the peak marker, and a blue rectangle shows the 95% confidence interval around the peak marker. The percentage of the total variance in the RIAIL population that can be explained by each QTL is shown above the QTL. The dotted vertical line represents the genomic position of *scb-1*.

We exposed a panel of 296 RIAILs (set 2 RIAILs, see Methods) to all eight chemotherapeutics at a selected concentration that both maximizes among-strain and minimizes within-strain phenotypic variation (File S2). Linkage mapping for all four traits for each of the eight drugs (total of 32 traits) identified 79 QTL from 31 traits (one trait had no significant QTL), several of which have been identified previously (Zdraljevic *et al.* 2017; Evans *et al.* 2018; Brady *et al.* 2019) (File S3, Figure S2). Strikingly, a QTL on the center of chromosome V was linked to variation in responses to all eight compounds (Figure 1). In all cases, the CB4856 allele on chromosome V is associated with greater resistance to the drug than the N2 allele (Figure S2, File S2, File S3). We previously identified this genomic interval as a QTL hotspot, defined as a region heavily enriched for toxin-response QTL (Evans *et al.* 2018). Because several of the chemotherapeutics share a similar mechanism of action, a single pleiotropic gene might underlie the observed QTL for multiple drugs.

**Figure 2:**
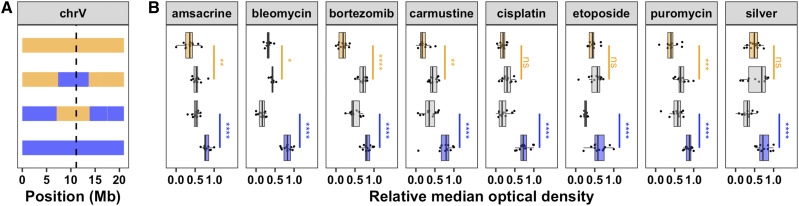
Near-isogenic lines validate the chromosome V QTL. (A) NIL genotypes on chromosome V are shown, colored orange (N2) and blue (CB4856). From top to bottom, strains are N2, ECA232, ECA1114, and CB4856. The dotted vertical line represents the location of *scb-1*. (B) NIL phenotypes in eight chemotherapeutics (12.5 µM amsacrine, 12.5 µM bleomycin, 2 µM bortezomib, 250 µM carmustine, 125 µM cisplatin, 62.5 µM etoposide, 300 µM puromycin, and 100 µM silver) are plotted as Tukey box plots with strain (y-axis) by relative median optical density (median.EXT, x-axis). Statistical significance was calculated for each strain pair (File S7). Significance of each strain compared to its parental strain (ECA232 to N2 and ECA1114 to CB4856) is shown above each strain pair and colored by the parent strain against which it was tested (ns = non-significant (p-value > 0.05); *, **, ***, and **** = significant (p-value < 0.05, 0.01, 0.001, or 0.0001, respectively).

In order to isolate and validate the effect of this QTL, we constructed reciprocal near-isogenic lines (NILs) by introgressing a genomic region on chromosome V from the resistant CB4856 strain into the sensitive N2 background and vice versa (File S4, File S5). We used a modified high-throughput assay (see Methods) to measure length and optical density of a population of animals grown in the presence of the drug for 48 hr (from larval stages L1 to L4). In this modified assay, less drug was required to observe the same phenotypic effect as before (Figure S3, File S6). Statistical significance was calculated in a pairwise manner for each strain (see Methods; File S7). For all eight chemotherapeutics tested, the strain with the N2 introgression was significantly more sensitive than its CB4856 parent and/or the strain with the CB4856 introgression was significantly more resistant than its N2 parent (Figure 2, File S6, File S7). These data confirm that one or more genetic variant(s) within this region on chromosome V cause increased drug sensitivities in N2.

### Expression QTL mapping identifies a hotspot on the center of chromosome V

Genetic variation can affect a phenotype most commonly through either modifications of the amino acid sequence that lead to altered protein function (or even loss of function) or changes in the expression level of the protein. In the latter case, measuring the intermediate phenotype (gene expression) can be useful in elucidating the mechanism by which genetic variation causes phenotypic variation. More specifically, cases with overlap between expression QTL (eQTL) and drug-response QTL suggest that a common variant could underlie both traits and provide evidence in support of causality for the candidate gene in question (Huang *et al.* 2015; Sasaki *et al.* 2018).

**Figure 3:**
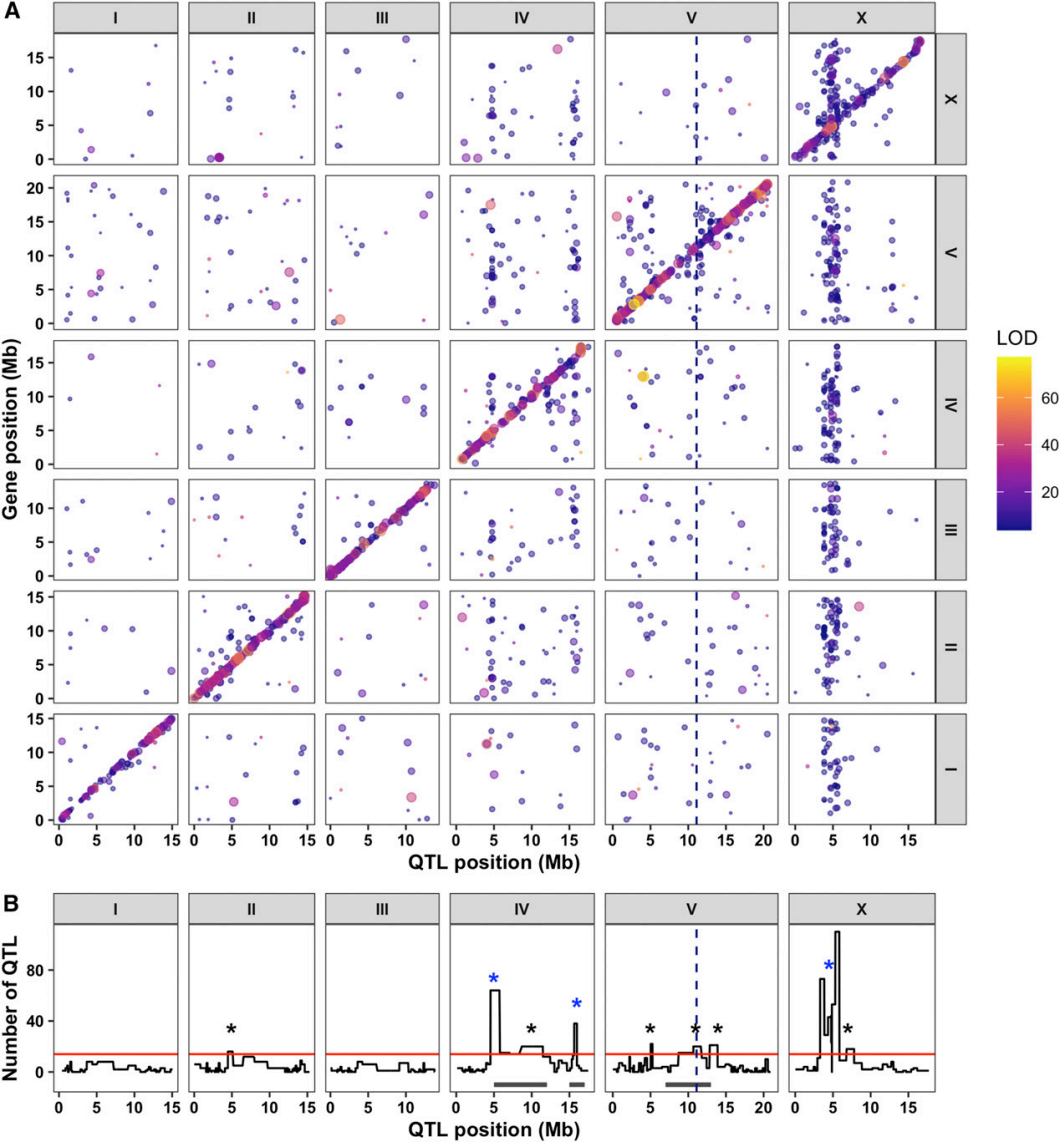
Expression QTL mapping identifies several hotspots. (A) The genomic locations of the eQTL peaks derived from linkage mapping using the set 1 RIAILs (x-axis) are plotted against the genomic locations of the probe (y-axis). The size of the point corresponds to the effect size of the QTL. eQTL are colored by the LOD score, increasing from purple to pink to yellow. The diagonal band represents local eQTL, and vertical bands represent eQTL hotspots. (B) Quantification of eQTL hotspots identified by overlapping distant eQTL. The number of distant eQTL (y-axis) in each 5 cM bin across the genome (x-axis) is shown. Bins above the red line are significant and marked with an asterisk. The bins with the blue asterisks are most significant and have been identified in a previous analysis. The dotted vertical line represents the genomic position of *scb-1*. Gray rectangles below the plot represent locations of the drug-response QTL hotspots previously identified.

To identify such cases of overlap between expression QTL and the drug-response QTL on chromosome V, we need genome-wide expression data for the RIAILs. In a previous study, expression of 15,888 probes were measured using microarrays for a panel of 208 RIAILs (set 1 RIAILs, see Methods) between N2 and CB4856 (Rockman and Kruglyak 2009) (File S8). This study used the variation in gene expression as a phenotypic trait to identify eQTL using linkage mapping with 1,455 variants (Rockman *et al.* 2010). They identified 2,309 eQTL and three regions with significantly clustered distant eQTL (eQTL hotspots), suggesting that these regions are pleiotropic, wherein one or more variant(s) are affecting expression of multiple genes. We recently performed whole-genome sequencing for these strains and identified 13,003 informative variants (Brady *et al.* 2019). Using this new set of variants, we re-analyzed the eQTL mapping by performing linkage mapping analysis for a selected 14,107 of the 15,888 probes without genetic variation in CB4856 (Andersen *et al.* 2014). We identified 2,540 eQTL associated with variation in expression of 2,196 genes (Figure 3A, File S9). These eQTL have relatively large effect sizes compared to the drug-response QTL. On average, each eQTL explains 23% of the phenotypic variance in gene expression among the RIAIL population. Half of the eQTL (50.2%; 1,276) mapped within 1 Mb of the gene whose expression was measured and were classified as local (see Methods) (Albert and Kruglyak 2015). The other half (49.7%; 1,264) were found distant from their respective gene, and over a third (37%; 940) were found on different chromosomes entirely. In general, eQTL effect sizes increased, max LOD scores decreased, and confidence intervals became smaller compared to the original mapping results (File S9). These differences and the additional eQTL observed between this analysis and the original are possibly caused by the integration of new genetic markers. Additionally, we found several differences in methodology between the current approach and the previous one. These differences include ignoring the population structure of the set 1 RIAILs, adding the forward-search marker-regression linkage mapping, and altering the linkage mapping method itself (see Methods, (Rockman *et al.* 2010)).

We noticed regions of the genome that appeared to be enriched for distant eQTL. We identified eQTL hotspots in a similar manner to the previous study (see Methods) and found a total of nine eQTL hotspots (Figure 3B, File S10). Six of the nine eQTL hotspots withstood more stringent filtering methods (see Methods), and three (left of chromosome IV, right of chromosome IV, and left of chromosome X) were the most significant. These three hotspots also overlap with the most significant eQTL hotspots in the previous study (Rockman *et al.* 2010). Notably, three of the eQTL hotspots (center of chromosome IV, right of chromosome IV, and center of chromosome V) overlap with the previously identified drug-response QTL hotspots on chromosomes IV and V (Figure 3B) (Evans *et al.* 2018). The overlap of these eQTL and drug-response QTL hotspots could provide strong candidate genes whose expression underlies the differences in nematode drug responses generally. Expression of one gene of interest, *scb-1*, has been previously implicated in response to bleomycin (Brady *et al.* 2019) and resides within the eQTL hotspot region on the center of chromosome V (File S10, Table S1). Although the exact mechanism of how *scb-1* responds to bleomycin is unknown, its putative hydrolase activity (Kelley *et al.* 2015; Zhang *et al.* 2018; Brady *et al.* 2019) suggests that it might act to break down chemotherapeutic compounds. These data suggest that variation in expression of *scb-1* and responses to these eight chemotherapeutics (including bleomycin) could be mechanistically linked through the metabolic breakdown of chemotherapeutic drugs.

### Mediation analysis suggests that scb-1 expression plays a role in responses to several chemotherapeutics

Mediation analysis seeks to explain the relationship between an independent and a dependent variable by including a third intermediary variable. We can use mediation analysis to understand how certain genetic variants on chromosome V (independent variable) affect drug responses (dependent variable) through differential gene expression of genes within the eQTL hotspot (mediator variable) (Figure S4). We measured brood size, animal length, and optical density in response to all eight chemotherapeutics in the set 1 RIAILs and performed linkage mapping for these traits (File S2, File S11, Figure S5). Although the power to detect QTL with these strains is lower than in our original mapping set (set 2 RIAILs; see Methods) (Andersen *et al.* 2015), we still identified overlapping QTL on chromosome V for half of the drugs tested (bleomycin, cisplatin, silver, and amsacrine) (Figure S5, File S11).

**Figure 4:**
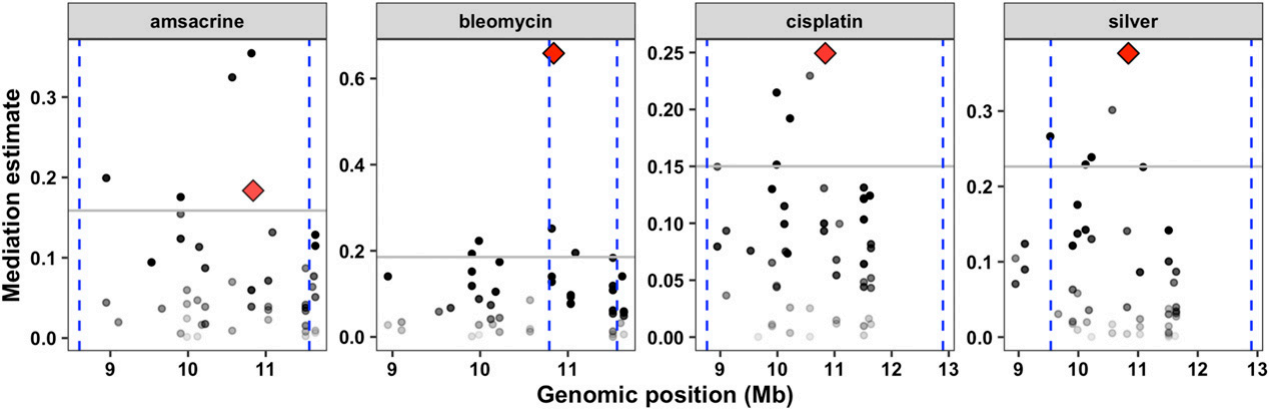
Mediation analysis for the eQTL hotspot on the center of chromosome V. Mediation estimates calculated as the indirect effect that differences in expression of each gene plays in the overall phenotype (y-axis) are plotted against the genomic position of the eQTL (x-axis) on chromosome V for 49 probes (including *scb-1* (red diamond)) that map to the chromosome V eQTL hotspot (set 1 RIAILs). A representative trait for each drug from the set 1 linkage mapping analysis are shown: amascrine (median.EXT), bleomycin (median.EXT), cisplatin (median.TOF), and silver (median.norm.EXT). The 90^th^ percentile of the distribution of mediation estimates for each trait are represented by the horizontal gray lines. The confidence intervals for the QTL (set 1 RIAILs) are shown with the vertical blue dotted lines. The confidence of the estimate increases (p-value decreases) as points become less transparent.

We calculated the effect that variation in expression of *scb-1* had on drug-response traits compared to the other 48 genes with an eQTL in the chromosome V eQTL hotspot using mediation analysis (see Methods). We estimated that the effect of expression variation of *scb-1* on bleomycin response is 0.65 (set 1 RIAILs, Figure 4, Figure S6, File S12). Moreover, out of all 49 genes with an eQTL in the region (Table S1), *scb-1* was a clear mediation score outlier. All of the remaining three chemotherapeutics with a QTL on the center of chromosome V in the set 1 RIAIL mapping showed moderate evidence of *scb-1* mediation, with *scb-1* falling well above the 90^th^ percentile of mediation estimates for all genes with an eQTL in this region (Figure 4, Figure S6, File S12). We further performed this mediation analysis on all 32 drug-response traits, regardless of the presence of a QTL in the set 1 RIAIL panel (Figure S6, File S12). Etoposide and puromycin also showed evidence of *scb-1* mediation. This *in silico* approach indicated that expression of *scb-1* might be an intermediate link between genetic variation on chromosome V and responses to several of the chemotherapeutic drugs tested.

### Expression of scb-1 affects responses to several chemotherapeutics that cause double-strand DNA breaks

**Figure 5:**
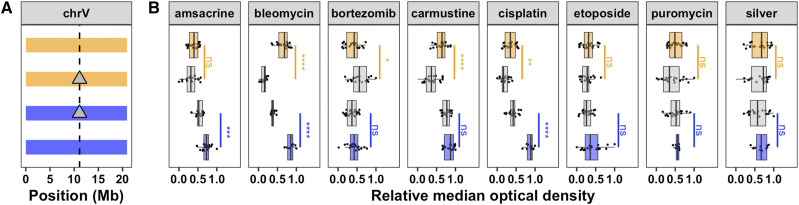
Testing the role of *scb-1* in drug responses. (A) Strain genotypes on chromosome V are shown, colored orange (N2) and blue (CB4856). From top to bottom, strains are N2, ECA1132, ECA1134, and CB4856. Deletion of *scb-1* is indicated by a gray triangle. The dotted vertical line represents the location of *scb-1*. (B) Phenotypes of strains in eight chemotherapeutics (12.5 µM amsacrine, 12.5 µM bleomycin, 2 µM bortezomib, 250 µM carmustine, 125 µM cisplatin, 62.5 µM etoposide, 300 µM puromycin, and 100 µM silver) are plotted as Tukey box plots with strain (y-axis) by relative median optical density (median.EXT, x-axis). Statistical significance was calculated for each strain pair (File S7). Significance of each deletion strain compared to its parental strain (ECA1132 to N2 and ECA1134 to CB4856) is shown above each strain pair and colored by the parent strain against which it was tested (ns = non-significant (p-value > 0.05); *, **, ***, and **** = significant (p-value < 0.05, 0.01, 0.001, or 0.0001, respectively).

To empirically test whether *scb-1* expression modulates the chromosome V QTL effect for each drug, we used the modified high-throughput assay (see Methods) to expose two independently derived strains with *scb-1* deletions (Brady *et al.* 2019) to each chemotherapeutic (Figure 5, Figure S7, File S13). Statistical significance was calculated in a pairwise manner for each strain (see Methods; File S7). Because RIAILs with the CB4856 allele on chromosome V express higher levels of *scb-1* than RIAILs with the N2 allele (File S8, File S9), we expect that loss of *scb-1* will cause increased drug sensitivity in the CB4856 background but might not have an effect in the N2 background. We validated the results of Brady *et al.* and confirmed that ablated *scb-1* expression causes hyper-sensitivity to bleomycin in both N2 and CB4856 (Figure 5, Figure S7, Figure S8 File S7, File S13). We also observed similarly increased sensitivity to cisplatin with *scb-1* deletions in both backgrounds. Furthermore, removing *scb-1* shows moderately increased sensitivity in the CB4856 background for amsacrine and in the N2 background for carmustine. The remaining four drugs did not show a significantly different phenotype between the parental N2 and CB4856 strains, suggesting these traits might be less reproducible or that *scb-1* variation does not underlie these drug differences. Overall, these results provide evidence for the pleiotropic effect of *scb-1*, which appears to mediate responses to at least four of the eight chemotherapeutic drugs.

## Discussion

In this study, we identified overlapping QTL on the center of chromosome V that influence sensitivities to eight chemotherapeutic drugs. Because five of these drugs are known to cause double-strand DNA breaks, we hypothesized that this genomic region might be pleiotropic – a single shared genetic variant affects the responses to each drug. Because this variant might affect drug responses by regulating gene expression levels, we looked for the co-existence of drug-response QTL and expression QTL on chromosome V. We identified 2,540 eQTL and nine eQTL hotspots, including a region on the center of chromosome V. We calculated the mediation effect of all 49 genes with an eQTL that maps to this hotspot region and identified *scb-1* as a candidate gene whose expression influences the responses to several chemotherapeutics. We used CRISPR-Cas9-mediated *scb-1* deletion strains to empirically validate the role of *scb-1* in the chemotherapeutic response. In addition to bleomycin (Brady *et al.* 2019), we discovered that responses to cisplatin, amsacrine, and carmustine are affected by *scb-1* expression. In this study, we found evidence that several overlapping QTL are representative of pleiotropy at the gene level and further elucidated the function of *scb-1* as a potential response to double-strand DNA break stress.

### Mediation of drug-response QTL using gene expression to identify causal genes

Mediation analysis often suggests potential candidate genes that underlie different traits (Huang *et al.* 2015; Sasaki *et al.* 2018) and could be applied to drug responses. Using *C. elegans* strains and high-throughput assays, we can rapidly validate hypotheses generated by mediation analysis. Three of the eight chemotherapeutics that map to an overlapping drug-response QTL and were potentially mediated by *scb-1* were validated using targeted deletion strains.

Although mediation analysis provided moderate evidence that expression of *scb-1* could also play a role in sensitivity to etoposide and puromycin, we observed no experimental evidence of this relationship. Additionally, we have evidence that expression of *scb-1* might mediate response to carmustine. However, mediation analysis disagrees. The discrepancy between the mediation analysis and validation of causality using targeted deletion strains could be partially explained by one of several possibilities. First, different traits were measured in each experiment. The mediation analysis used traits measured over 96 hr of growth in drug conditions spanning two generations, but the causality test used traits measured over 48 hr of growth in drug conditions within one generation. Second, the precision of our mediation estimates was likely reduced by the poor quality drug traits for the set 1 RIAIL panel (Andersen *et al.* 2015). Indeed, bortezomib, carmustine, etoposide, and puromycin did not map to the center of chromosome V using the set 1 RIAILs (Figure S5). Expression data for the set 2 RIAIL panel would likely generate more accurate mediation estimates, especially if data were collected using RNA sequencing to avoid the inherent reference bias of microarray data (Zhao *et al.* 2014). Third, our mediation analysis was performed using expression data collected in control conditions and phenotype data collected in drug conditions. This analysis will only provide evidence of mediation if the baseline expression differences affect an individual’s response to the drug. Collecting expression data from drug-treated nematodes could help us learn more about how gene expression varies in response to treatment with the chemotherapeutic. Finally, as we only directly assessed the complete loss of *scb-1* in drug sensitivity, it is still possible that reduction of function (or change in function) caused by a single nucleotide variant or other structural variation in CB4856 could validate the role of *scb-1* in responses to these drugs.

This study demonstrates the power of pairing genome-wide linkage mapping of gene expression and drug response data using simple colocalization as well as more complex mediation analysis techniques. In addition to providing a resource for candidate gene prioritization within a QTL interval, mediation analysis can help to identify the mechanism by which genetic variation causes phenotypic differences. This type of approach could be even more powerful using genome-wide association (GWA) where the lower linkage disequilibrium between variants also has smaller confidence intervals in some genomic regions. Smaller intervals have fewer spurious overlapping eQTL, which could help to narrow the list of candidate genes. Although mediation analysis is only effective if a change in expression is observed and might not be useful for identifying effects from protein-coding variation, many current studies show that the majority of genetic variants associated with complex traits lie in regulatory regions (Hindorff *et al.* 2009). Whole-genome expression analysis could provide the missing link to the identification of causal genes underlying complex traits.

### New evidence for the pleiotropic function of scb-1

We identified eight chemotherapeutics with a QTL that mapped to a genomic region defined as a QTL hotspot on the center of chromosome V (Evans *et al.* 2018). Multiple genes in close proximity, each regulating an aspect of cellular growth and fitness, might underlie each QTL independently. Alternatively, genetic variation within a single gene might regulate responses to multiple (or all) of the eight drugs tested, particularly if the gene is involved in drug transport or metabolism or if the drug mechanisms of action were shared (*e.g.*, repair of double-strand DNA breaks). Expression of *scb-1*, a gene previously implicated in modulating responses to bleomycin, was found to reduce sensitivity to half of the drugs tested. This pleiotropic effect of *scb-1* provides new evidence for the function of the gene and possible molecular mechanisms underlying nematode drug responses. It is hypothesized that SCB-1 might function as a hydrolase that metabolizes compounds like bleomycin (Brady *et al.* 2019) or somehow plays a role in the nematode stress response (Riedel *et al.* 2013). Both hypotheses are consistent with our data, explaining why nematodes with low expression of *scb-1* are highly sensitive to the compound. Furthermore, all four of these chemotherapeutics, whose responses are mediated by expression of *scb-1*, are known to cause double-strand DNA breaks (Dorr 1992; Ketron *et al.* 2012; Dasari and Tchounwou 2014; Nikolova *et al.* 2017). Although the results for bortezomib, puromycin, and silver were inconclusive, we found no clear evidence that expression of *scb-1* dictates their responses. Together, these data suggest a potential role for *scb-1* specifically in response to stress induced by double-strand DNA breaks. However, the lack of sensitivity in etoposide, which also causes double-strand DNA breaks (Montecucco *et al.* 2015), indicates that this response might be more complex.

The exact variant that causes the differential expression of *scb-1* is still unknown. Importantly, *scb-1* lies within an eQTL hotspot region where it is hypothesized that genetic variation at a single locus might regulate expression of the 49 genes with an eQTL in this region. It is possible that the same causal variant that regulates expression of *scb-1* could also underlie the QTL for the remaining four chemotherapeutics through differential expression of other genes. For example, mediation analysis for both bortezomib and etoposide indicated that expression variation of a dehydrogenase (*D1054.8*) may underlie their differential responses (File S12). Alternatively, the causal variants underlying these drug-response QTL might be distinct but physically linked in the genome. This result would suggest a cluster of genes essential for the nematode drug response. Overall, our study highlights the power of using mediation analysis to connect gene expression to organismal traits and describes a novel function for the pleiotropic gene *scb-1*.

## References

[bib1] Albert F. W., Bloom J. S., Siegel J., Day L., and Kruglyak L., 2018 Genetics of trans-regulatory variation in gene expression. eLife 7 10.7554/eLife.35471 PMC6072440 30014850

[bib2] Albert F. W., and Kruglyak L., 2015 The role of regulatory variation in complex traits and disease. Nat. Rev. Genet. 16: 197–212. 10.1038/nrg3891 25707927

[bib3] Andersen E. C., Bloom J. S., Gerke J. P., and Kruglyak L., 2014 A variant in the neuropeptide receptor npr-1 is a major determinant of Caenorhabditis elegans growth and physiology. PLoS Genet. 10: e1004156 10.1371/journal.pgen.1004156 24586193 PMC3937155

[bib4] Andersen E. C., Shimko T. C., Crissman J. R., Ghosh R., Bloom J. S., 2015 A Powerful New Quantitative Genetics Platform, Combining Caenorhabditis elegans High-Throughput Fitness Assays with a Large Collection of Recombinant Strains. G3 (Bethesda) 5: 911–920. 10.1534/g3.115.017178 25770127 PMC4426375

[bib5] Azzam M. E., and Algranati I. D., 1973 Mechanism of puromycin action: fate of ribosomes after release of nascent protein chains from polysomes. Proc. Natl. Acad. Sci. USA 70: 3866–3869. 10.1073/pnas.70.12.3866 4590173 PMC427346

[bib6] Balla K. M., Andersen E. C., Kruglyak L., and Troemel E. R., 2015 A wild C. elegans strain has enhanced epithelial immunity to a natural microsporidian parasite. PLoS Pathog. 11: e1004583 10.1371/journal.ppat.1004583 25680197 PMC4334554

[bib7] Bates D., Mächler M., Bolker B., and Walker S., 2014 Fitting Linear Mixed-Effects Models using lme4. arXiv. 10.18637/jss.v067.i01

[bib8] Bendesky A., and Bargmann C. I., 2011 Genetic contributions to behavioural diversity at the gene-environment interface. Nat. Rev. Genet. 12: 809–820. 10.1038/nrg3065 22064512

[bib9] Bendesky A., Pitts J., Rockman M. V., Chen W. C., Tan M.-W., 2012 Long-range regulatory polymorphisms affecting a GABA receptor constitute a quantitative trait locus (QTL) for social behavior in Caenorhabditis elegans. PLoS Genet. 8: e1003157 10.1371/journal.pgen.1003157 23284308 PMC3527333

[bib10] Bendesky A., Tsunozaki M., Rockman M. V., Kruglyak L., and Bargmann C. I., 2011 Catecholamine receptor polymorphisms affect decision-making in C. elegans. Nature 472: 313–318. 10.1038/nature09821 21412235 PMC3154120

[bib11] Bloom J. S., Ehrenreich I. M., Loo W. T., Lite T.-L. V., and Kruglyak L., 2013 Finding the sources of missing heritability in a yeast cross. Nature 494: 234–237. 10.1038/nature11867 23376951 PMC4001867

[bib12] Borrello M. G., Degl’Innocenti D., and Pierotti M. A., 2008 Inflammation and cancer: the oncogene-driven connection. Cancer Lett. 267: 262–270. 10.1016/j.canlet.2008.03.060 18502035

[bib13] Boyd W. A., Smith M. V., and Freedman J. H., 2012 Caenorhabditis elegans as a model in developmental toxicology. Methods Mol. Biol. 889: 15–24. 10.1007/978-1-61779-867-2_3 22669657 PMC3513774

[bib14] Brady S. C., Zdraljevic S., Bisaga K. W., Tanny R. E., Cook D. E., 2019 A Novel Gene Underlies Bleomycin-Response Variation in Caenorhabditis elegans. Genetics 212: 1453–1468. 10.1534/genetics.119.302286 31171655 PMC6707474

[bib15] Breitling R., Li Y., Tesson B. M., Fu J., Wu C., 2008 Genetical genomics: spotlight on QTL hotspots. PLoS Genet. 4: e1000232 10.1371/journal.pgen.1000232 18949031 PMC2563687

[bib16] Brem R. B., Yvert G., Clinton R., and Kruglyak L., 2002 Genetic dissection of transcriptional regulation in budding yeast. Science 296: 752–755. 10.1126/science.1069516 11923494

[bib17] Broman K. W., Wu H., Sen S., and Churchill G. A., 2003 R/qtl: QTL mapping in experimental crosses. Bioinformatics 19: 889–890. 10.1093/bioinformatics/btg112 12724300

[bib18] Brown E. B., Layne J. E., Zhu C., Jegga A. G., and Rollmann S. M., 2013 Genome-wide association mapping of natural variation in odour-guided behaviour in Drosophila. Genes Brain Behav. 12: 503–515. 10.1111/gbb.12048 23682951

[bib19] Chesmore K., Bartlett J., and Williams S. M., 2018 The ubiquity of pleiotropy in human disease. Hum. Genet. 137: 39–44. 10.1007/s00439-017-1854-z 29164333

[bib20] Cook D. E., Zdraljevic S., Roberts J. P., and Andersen E. C., 2017 CeNDR, the Caenorhabditis elegans natural diversity resource. Nucleic Acids Res. 45: D650–D657. 10.1093/nar/gkw893 27701074 PMC5210618

[bib21] Cubillos F. A., Billi E., Zörgö E., Parts L., Fargier P., 2011 Assessing the complex architecture of polygenic traits in diverged yeast populations. Mol. Ecol. 20: 1401–1413. 10.1111/j.1365-294X.2011.05005.x 21261765

[bib22] Dasari S., and Tchounwou P. B., 2014 Cisplatin in cancer therapy: molecular mechanisms of action. Eur. J. Pharmacol. 740: 364–378. 10.1016/j.ejphar.2014.07.025 25058905 PMC4146684

[bib23] Doroszuk A., Snoek L. B., Fradin E., Riksen J., and Kammenga J., 2009 A genome-wide library of CB4856/N2 introgression lines of Caenorhabditis elegans. Nucleic Acids Res. 37: e110 10.1093/nar/gkp528 19542186 PMC2760803

[bib24] Dorr R. T., 1992 Bleomycin pharmacology: mechanism of action and resistance, and clinical pharmacokinetics. Semin. Oncol. 19: 3–8. 1384141

[bib25] El‐Assal S. E. D., Alonso‐Blanco C., Hanhart C. J., and Koornneef M., 2004 Pleiotropic Effects of the Arabidopsis Cryptochrome 2 Allelic Variation Underlie Fruit Trait‐Related QTL. Plant Biol. 6: 370–374. 10.1055/s-2004-820890 15248119

[bib26] Evans K. S., Brady S. C., Bloom J. S., Tanny R. E., Cook D. E., 2018 Shared Genomic Regions Underlie Natural Variation in Diverse Toxin Responses. Genetics 210: 1509–1525. 10.1534/genetics.118.301311 30341085 PMC6283156

[bib27] Fisher R. A.1930 The genetical theory of natural selection. The Clarendon Press.

[bib28] Fusari C. M., Kooke R., Lauxmann M. A., Annunziata M. G., Enke B., 2017 Genome-Wide Association Mapping Reveals That Specific and Pleiotropic Regulatory Mechanisms Fine-Tune Central Metabolism and Growth in Arabidopsis. Plant Cell 29: 2349–2373. 10.1105/tpc.17.00232 28954812 PMC5774568

[bib29] García-González A. P., Ritter A. D., Shrestha S., Andersen E. C., Yilmaz L. S., 2017 Bacterial Metabolism Affects the C. elegans Response to Cancer Chemotherapeutics. Cell 169: 431–441.e8. 10.1016/j.cell.2017.03.046 28431244 PMC5484065

[bib30] Ghosh R., Andersen E. C., Shapiro J. A., Gerke J. P., and Kruglyak L., 2012 Natural variation in a chloride channel subunit confers avermectin resistance in C. elegans. Science 335: 574–578. 10.1126/science.1214318 22301316 PMC3273849

[bib31] Glater E. E., Rockman M. V., and Bargmann C. I., 2014 Multigenic natural variation underlies Caenorhabditis elegans olfactory preference for the bacterial pathogen Serratia marcescens. G3 (Bethesda) 4: 265–276. 10.1534/g3.113.008649 24347628 PMC3931561

[bib32] Gratten J., and Visscher P. M., 2016 Genetic pleiotropy in complex traits and diseases: implications for genomic medicine. Genome Med. 8: 78 10.1186/s13073-016-0332-x 27435222 PMC4952057

[bib33] Gutteling E. W., Doroszuk A., Riksen J. A. G., Prokop Z., Reszka J., 2007 a Environmental influence on the genetic correlations between life-history traits in Caenorhabditis elegans. Heredity 98: 206–213. 10.1038/sj.hdy.6800929 17203010

[bib34] Gutteling E. W., Riksen J. A. G., Bakker J., and Kammenga J. E., 2007 b Mapping phenotypic plasticity and genotype-environment interactions affecting life-history traits in Caenorhabditis elegans. Heredity 98: 28–37. 10.1038/sj.hdy.6800894 16955112

[bib35] Hasin-Brumshtein Y., Khan A. H., Hormozdiari F., Pan C., Parks B. W., 2016 Hypothalamic transcriptomes of 99 mouse strains reveal trans eQTL hotspots, splicing QTLs and novel non-coding genes. eLife 5 10.7554/eLife.15614 PMC5053804 27623010

[bib36] Hindorff L. A., Sethupathy P., Junkins H. A., Ramos E. M., Mehta J. P., 2009 Potential etiologic and functional implications of genome-wide association loci for human diseases and traits. Proc. Natl. Acad. Sci. USA 106: 9362–9367. 10.1073/pnas.0903103106 19474294 PMC2687147

[bib37] Huang Y.-T., Liang L., Moffatt M. F., Cookson W. O. C. M., and Lin X., 2015 iGWAS: Integrative Genome-Wide Association Studies of Genetic and Genomic Data for Disease Susceptibility Using Mediation Analysis. Genet. Epidemiol. 39: 347–356. 10.1002/gepi.21905 25997986 PMC4544880

[bib38] Jerison E. R., Kryazhimskiy S., Mitchell J. K., Bloom J. S., Kruglyak L., 2017 Genetic variation in adaptability and pleiotropy in budding yeast. eLife 6 10.7554/eLife.27167 PMC5580887 28826486

[bib39] Kammenga J. E., Doroszuk A., Riksen J. A. G., Hazendonk E., Spiridon L., 2007 A Caenorhabditis elegans wild type defies the temperature-size rule owing to a single nucleotide polymorphism in tra-3. PLoS Genet. 3: e34 10.1371/journal.pgen.0030034 17335351 PMC1808073

[bib40] Kaplan A., Akalin Ciftci G., and Kutlu H. M., 2016 Cytotoxic, anti-proliferative and apoptotic effects of silver nitrate against H-ras transformed 5RP7. Cytotechnology 68: 1727–1735. 10.1007/s10616-015-9922-5 26499861 PMC5023546

[bib41] Kelley L. A., Mezulis S., Yates C. M., Wass M. N., and Sternberg M. J. E., 2015 The Phyre2 web portal for protein modeling, prediction and analysis. Nat. Protoc. 10: 845–858. 10.1038/nprot.2015.053 25950237 PMC5298202

[bib42] Ketron A. C., Denny W. A., Graves D. E., and Osheroff N., 2012 Amsacrine as a topoisomerase II poison: importance of drug-DNA interactions. Biochemistry 51: 1730–1739. 10.1021/bi201159b 22304499 PMC3289736

[bib43] Keurentjes J. J. B., Fu J., Terpstra I. R., Garcia J. M., van den Ackerveken G., 2007 Regulatory network construction in Arabidopsis by using genome-wide gene expression quantitative trait loci. Proc. Natl. Acad. Sci. USA 104: 1708–1713. 10.1073/pnas.0610429104 17237218 PMC1785256

[bib44] Leamy L. J., Elo K., Nielsen M. K., Thorn S. R., Valdar W., 2014 Quantitative trait loci for energy balance traits in an advanced intercross line derived from mice divergently selected for heat loss. PeerJ 2: e392 10.7717/peerj.392 24918027 PMC4045330

[bib45] Lee D., Yang H., Kim J., Brady S., Zdraljevic S., 2017 The genetic basis of natural variation in a phoretic behavior. Nat. Commun. 8: 273 10.1038/s41467-017-00386-x 28819099 PMC5561207

[bib46] Li Y., Alvarez O. A., Gutteling E. W., Tijsterman M., Fu J., 2006 Mapping determinants of gene expression plasticity by genetical genomics in C. elegans. PLoS Genet. 2: e222 10.1371/journal.pgen.0020222 17196041 PMC1756913

[bib47] Lin C. H. S., Chen J., Ziman B., Marshall S., Maizel J., 2014 Endostatin and kidney fibrosis in aging: a case for antagonistic pleiotropy? Am. J. Physiol. Heart Circ. Physiol. 306: H1692–H1699. 10.1152/ajpheart.00064.2014 24727495 PMC4059979

[bib48] MacKinnon D. P., Fairchild A. J., and Fritz M. S., 2007 Mediation analysis. Annu. Rev. Psychol. 58: 593–614. 10.1146/annurev.psych.58.110405.085542 16968208 PMC2819368

[bib49] McGrath P. T., Rockman M. V., Zimmer M., Jang H., Macosko E. Z., 2009 Quantitative mapping of a digenic behavioral trait implicates globin variation in C. elegans sensory behaviors. Neuron 61: 692–699. 10.1016/j.neuron.2009.02.012 19285466 PMC2772867

[bib50] McGuigan K., Collet J. M., McGraw E. A., Ye Y. H., Allen S. L., 2014 The nature and extent of mutational pleiotropy in gene expression of male Drosophila serrata. Genetics 196: 911–921. 10.1534/genetics.114.161232 24402375 PMC3948815

[bib51] McKay J. K., Richards J. H., and Mitchell-Olds T., 2003 Genetics of drought adaptation in Arabidopsis thaliana: I. Pleiotropy contributes to genetic correlations among ecological traits. Mol. Ecol. 12: 1137–1151. 10.1046/j.1365-294X.2003.01833.x 12694278

[bib52] Montecucco A., Zanetta F., and Biamonti G., 2015 Molecular mechanisms of etoposide. EXCLI J. 14: 95–108. 26600742 10.17179/excli2015-561 PMC4652635

[bib53] Nikolova T., Roos W. P., Krämer O. H., Strik H. M., and Kaina B., 2017 Chloroethylating nitrosoureas in cancer therapy: DNA damage, repair and cell death signaling. Biochim. Biophys. Acta 1868: 29–39. 10.1016/j.bbcan.2017.01.004 28143714

[bib54] Orr H. A., 2000 Adaptation and the cost of complexity. Evolution 54: 13–20. 10.1111/j.0014-3820.2000.tb00002.x 10937178

[bib55] Paaby A. B., and Rockman M. V., 2013 The many faces of pleiotropy. Trends Genet. 29: 66–73. 10.1016/j.tig.2012.10.010 23140989 PMC3558540

[bib56] Pavlides J. M. W., Zhu Z., Gratten J., McRae A. F., Wray N. R., 2016 Predicting gene targets from integrative analyses of summary data from GWAS and eQTL studies for 28 human complex traits. Genome Med. 8: 84 10.1186/s13073-016-0338-4 27506385 PMC4979185

[bib57] Peltier E., Friedrich A., Schacherer J., and Marullo P., 2019 Quantitative Trait Nucleotides Impacting the Technological Performances of Industrial Saccharomyces cerevisiae Strains. Front. Genet. 10: 683 10.3389/fgene.2019.00683 31396264 PMC6664092

[bib58] Piperdi B., Ling Y.-H., Liebes L., Muggia F., and Perez-Soler R., 2011 Bortezomib: understanding the mechanism of action. Mol. Cancer Ther. 10: 2029–2030. 10.1158/1535-7163.MCT-11-0745 22072812

[bib59] R Core Team, 2017 R: A Language and Environment for Statistical Computing. R Foundation for Statistical Computing, Vienna, Austria. https://www.R-project.org/.

[bib60] Reddy K. C., Andersen E. C., Kruglyak L., and Kim D. H., 2009 A polymorphism in npr-1 is a behavioral determinant of pathogen susceptibility in C. elegans. Science 323: 382–384. 10.1126/science.1166527 19150845 PMC2748219

[bib61] Riedel C. G., Dowen R. H., Lourenco G. F., Kirienko N. V., Heimbucher T., 2013 DAF-16 employs the chromatin remodeller SWI/SNF to promote stress resistance and longevity. Nat. Cell Biol. 15: 491–501. 10.1038/ncb2720 23604319 PMC3748955

[bib62] Rockman M. V., and Kruglyak L., 2009 Recombinational landscape and population genomics of Caenorhabditis elegans. PLoS Genet. 5: e1000419 10.1371/journal.pgen.1000419 19283065 PMC2652117

[bib63] Rockman M. V., Skrovanek S. S., and Kruglyak L., 2010 Selection at linked sites shapes heritable phenotypic variation in C. elegans. Science 330: 372–376. 10.1126/science.1194208 20947766 PMC3138179

[bib64] Rodriguez M., Snoek L. B., Riksen J. A. G., Bevers R. P., and Kammenga J. E., 2012 Genetic variation for stress-response hormesis in C. elegans lifespan. Exp. Gerontol. 47: 581–587. 10.1016/j.exger.2012.05.005 22613270

[bib65] Sasaki E., Frommlet F., and Nordborg M., 2018 GWAS with Heterogeneous Data: Estimating the Fraction of Phenotypic Variation Mediated by Gene Expression Data. G3 (Bethesda) 8: 3059–3068. 10.1534/g3.118.200571 30068524 PMC6118313

[bib66] Schmid T., Snoek L. B., Fröhli E., van der Bent M. L., Kammenga J., 2015 Systemic Regulation of RAS/MAPK Signaling by the Serotonin Metabolite 5-HIAA. PLoS Genet. 11: e1005236 10.1371/journal.pgen.1005236 25978500 PMC4433219

[bib67] Seidel H. S., Ailion M., Li J., van Oudenaarden A., Rockman M. V., 2011 A novel sperm-delivered toxin causes late-stage embryo lethality and transmission ratio distortion in C. elegans. PLoS Biol. 9: e1001115 10.1371/journal.pbio.1001115 21814493 PMC3144186

[bib68] Seidel H. S., Rockman M. V., and Kruglyak L., 2008 Widespread genetic incompatibility in C. elegans maintained by balancing selection. Science 319: 589–594. 10.1126/science.1151107 18187622 PMC2421010

[bib69] Shimko T. C., and Andersen E. C., 2014 COPASutils: an R package for reading, processing, and visualizing data from COPAS large-particle flow cytometers. PLoS One 9: e111090 10.1371/journal.pone.0111090 25329171 PMC4203834

[bib70] Singh K. D., Roschitzki B., Snoek L. B., Grossmann J., Zheng X., 2016 Natural Genetic Variation Influences Protein Abundances in C. elegans Developmental Signalling Pathways. PLoS One 11: e0149418 10.1371/journal.pone.0149418 26985669 PMC4795773

[bib71] Sivakumaran S., Agakov F., Theodoratou E., Prendergast J. G., Zgaga L., 2011 Abundant pleiotropy in human complex diseases and traits. Am. J. Hum. Genet. 89: 607–618. 10.1016/j.ajhg.2011.10.004 22077970 PMC3213397

[bib72] Snoek L. B., Orbidans H. E., Stastna J. J., Aartse A., Rodriguez M., 2014 Widespread genomic incompatibilities in Caenorhabditis elegans. G3 (Bethesda) 4: 1813–1823. 10.1534/g3.114.013151 25128438 PMC4199689

[bib73] Sterken M. G., Snoek L. B., Kammenga J. E., and Andersen E. C., 2015 The laboratory domestication of Caenorhabditis elegans. Trends Genet. 31: 224–231. 10.1016/j.tig.2015.02.009 25804345 PMC4417040

[bib74] Tingley D., Yamamoto T., Hirose K., Keele L., and Imai K., 2014 mediation: R Package for Causal Mediation Analysis. Journal of Statistical Software. Articles 59: 1–38.

[bib75] Tyler A. L., Crawford D. C., and Pendergrass S. A., 2016 The detection and characterization of pleiotropy: discovery, progress, and promise. Brief. Bioinform. 17: 13–22. 10.1093/bib/bbv050 26223525

[bib76] Viñuela A., Snoek L. B., Riksen J. A. G., and Kammenga J. E., 2010 Genome-wide gene expression regulation as a function of genotype and age in C. elegans. Genome Res. 20: 929–937. 10.1101/gr.102160.109 20488933 PMC2892094

[bib77] Wagner G. P., and Zhang J., 2011 The pleiotropic structure of the genotype-phenotype map: the evolvability of complex organisms. Nat. Rev. Genet. 12: 204–213. 10.1038/nrg2949 21331091

[bib78] White J. K., Gerdin A.-K., Karp N. A., Ryder E., Buljan M., 2013 Genome-wide generation and systematic phenotyping of knockout mice reveals new roles for many genes. Cell 154: 452–464. 10.1016/j.cell.2013.06.022 23870131 PMC3717207

[bib79] Zamanian M., Cook D. E., Zdraljevic S., Brady S. C., Lee D., 2018 a Discovery of genomic intervals that underlie nematode responses to benzimidazoles. PLoS Negl. Trop. Dis. 12: e0006368 10.1371/journal.pntd.0006368 29601575 PMC5895046

[bib80] Zamanian M., Cook D. E., Zdraljevic S., Brady S. C., Lee D., 2018 b Discovery of genomic intervals that underlie nematode responses to benzimidazoles. PLoS Negl. Trop. Dis. 12: e0006368 10.1371/journal.pntd.0006368 29601575 PMC5895046

[bib81] Zdraljevic S., Fox B. W., Strand C., Panda O., Tenjo F. J., 2019 Natural variation in C. elegans arsenic toxicity is explained by differences in branched chain amino acid metabolism. eLife 8 10.7554/eLife.40260 PMC6453569 30958264

[bib82] Zdraljevic S., Strand C., Seidel H. S., Cook D. E., Doench J. G., 2017 Natural variation in a single amino acid substitution underlies physiological responses to topoisomerase II poisons. PLoS Genet. 13: e1006891 10.1371/journal.pgen.1006891 28700616 PMC5529024

[bib83] Zhang L., Li L., Yan L., Ming Z., Jia Z., 2018 Structural and Biochemical Characterization of Endoribonuclease Nsp15 Encoded by Middle East Respiratory Syndrome Coronavirus. J. Virol. 92 10.1128/JVI.00893-18 PMC6206473 30135128

[bib84] Zhao S., Fung-Leung W.-P., Bittner A., Ngo K., and Liu X., 2014 Comparison of RNA-Seq and microarray in transcriptome profiling of activated T cells. PLoS One 9: e78644 10.1371/journal.pone.0078644 24454679 PMC3894192

